# Supplementary material for: Resolving the full spectrum of human genome variation using Linked-Reads
Source: Genome Res. 2019 Apr;29(4):635–45. doi: 10.1101/gr.234443.118 (PMC6442396; doi:10.1101/gr.234443.118)
Supplement: Supplemental Material [file supp_gr.234443.118_Supplemental_longranger-2.2.zip › longranger-2.2.tar.bz2!/lr2.2-public/lib/stan/cmdstan/stan/doc/api/html/arg__engine_8hpp_source.html]

Stan: src/stan/gm/arguments/arg\_engine.hpp Source File


|  |  |
| --- | --- |
|  | Stan  2.5.0  probability, sampling & optimization |


- Main Page
- Related Pages
- Namespaces
- Classes
- Files

- File List
- File Members

All Classes Namespaces Files Functions Variables Typedefs Enumerations Enumerator Friends Macros Pages

- src
- stan
- gm
- arguments

arg\_engine.hpp

Go to the documentation of this file.

1 #ifndef STAN\_\_GM\_\_ARGUMENTS\_\_ENGINE\_\_HPP

2 #define STAN\_\_GM\_\_ARGUMENTS\_\_ENGINE\_\_HPP

3

4 #include <stan/gm/arguments/list\_argument.hpp>

5

6 #include <stan/gm/arguments/arg\_static.hpp>

7 #include <stan/gm/arguments/arg\_nuts.hpp>

8

9 namespace stan {

10

11  namespace gm {

12

13  class arg\_engine: public list\_argument {

14

15  public:

16

17  arg\_engine() {

18

19  \_name = "engine";

20  \_description = "Engine for Hamiltonian Monte Carlo";

21

22  \_values.push\_back(new arg\_static());

23  \_values.push\_back(new arg\_nuts());

24

25  \_default\_cursor = 1;

26  \_cursor = \_default\_cursor;

27

28  }

29

30  };

31

32  } // gm

33

34 } // stan

35

36 #endif

37

arg\_nuts.hpp

stan::gm::arg\_nuts

**Definition:** arg\_nuts.hpp:12

stan::gm::list\_argument::\_default\_cursor

int \_default\_cursor

**Definition:** list\_argument.hpp:183

list\_argument.hpp

stan::gm::list\_argument::\_values

std::vector< argument \* > \_values

**Definition:** list\_argument.hpp:185

stan::gm::list\_argument

**Definition:** list\_argument.hpp:12

stan::gm::arg\_engine

**Definition:** arg\_engine.hpp:13

stan::gm::arg\_engine::arg\_engine

arg\_engine()

**Definition:** arg\_engine.hpp:17

stan::gm::arg\_static

**Definition:** arg\_static.hpp:12

stan::gm::argument::\_name

std::string \_name

**Definition:** argument.hpp:76

arg\_static.hpp

stan::gm::list\_argument::\_cursor

int \_cursor

**Definition:** list\_argument.hpp:182

stan::gm::argument::\_description

std::string \_description

**Definition:** argument.hpp:77


---

[ Stan Home Page ]

*© 2011–2014,
Stan Development Team.*
